# The Marine Polysaccharide Ulvan Confers Potent Osteoinductive Capacity to PCL-Based Scaffolds for Bone Tissue Engineering Applications

**DOI:** 10.3390/ijms22063086

**Published:** 2021-03-17

**Authors:** Stefanos Kikionis, Efstathia Ioannou, Eleni Aggelidou, Leto-Aikaterini Tziveleka, Efterpi Demiri, Athina Bakopoulou, Spiros Zinelis, Aristeidis Kritis, Vassilios Roussis

**Affiliations:** 1 Section of Pharmacognosy and Chemistry of Natural Products, Department of Pharmacy, National and Kapodistrian University of Athens, Panepistimiopolis Zografou, 15771 Athens, Greece; skikionis@pharm.uoa.gr (S.K.); eioannou@pharm.uoa.gr (E.I.); ltziveleka@pharm.uoa.gr (L.-A.T.); 2 cGMP Regenerative Medicine Facility, Department of Physiology and Pharmacology, School of Medicine, Faculty of Health Sciences, Aristotle University of Thessaloniki, 54124 Thessaloniki, Greece; angelide@auth.gr (E.A.); abakopoulou@dent.auth.gr (A.B.); kritis@auth.gr (A.K.); 3 Department of Physiology and Pharmacology, School of Medicine, Faculty of Health Sciences, Aristotle University of Thessaloniki, 54124 Thessaloniki, Greece; 4 Department of Plastic Surgery, School of Medicine, Faculty of Health Sciences, Papageorgiou Hospital, Aristotle University of Thessaloniki, 54124 Thessaloniki, Greece; demirie@auth.gr; 5 Laboratory of Prosthodontics, School of Dentistry, Aristotle University of Thessaloniki, 54124 Thessaloniki, Greece; 6 Department of Biomaterials, School of Dentistry, National and Kapodistrian University of Athens, 11527 Athens, Greece; szinelis@dent.uoa.gr

**Keywords:** ulvan, hybrid scaffolds, mesenchymal stem cells, osteoinductive capacity, osteogenic differentiation, bone tissue engineering

## Abstract

Hybrid composites of synthetic and natural polymers represent materials of choice for bone tissue engineering. Ulvan, a biologically active marine sulfated polysaccharide, is attracting great interest in the development of novel biomedical scaffolds due to recent reports on its osteoinductive properties. Herein, a series of hybrid polycaprolactone scaffolds containing ulvan either alone or in blends with κ-carrageenan and chondroitin sulfate was prepared and characterized. The impact of the preparation methodology and the polysaccharide composition on their morphology, as well as on their mechanical, thermal, water uptake and porosity properties was determined, while their osteoinductive potential was investigated through the evaluation of cell adhesion, viability, and osteogenic differentiation of seeded human adipose-derived mesenchymal stem cells. The results verified the osteoinductive ability of ulvan, showing that its incorporation into the polycaprolactone matrix efficiently promoted cell attachment and viability, thus confirming its potential in the development of biomedical scaffolds for bone tissue regeneration applications.

## 1. Introduction

Bone tissue engineering is an emerging interdisciplinary field, combining biology, materials science, and engineering principles and techniques for the regeneration or restoration of damaged bone [1]. Bone is a hierarchically structured composite material with a composition of approximately 65 wt.% mineral phase, 25 wt.% organic matter, and 10 wt.% water [2]. Bone extracellular matrix (ECM) is composed of organic constituents, including collagen and non-collagenous proteins, and inorganic minerals, such as nano-hydroxyapatite, and serves as a frame for cell adhesion, migration, proliferation and differentiation [3]. The application of biomaterial scaffolds that mimic the ECM providing a bone-like microenviroment represents the most classic approach for bone regeneration [4,5]. Towards successful bone tissue engineering, several methods and materials have been applied for the development of composite 3D porous scaffolds that mimic the in vivo cellular environment [6,7].

Ideally, bone tissue engineering scaffolds should be biocompatible, biodegradable, non-toxic with high porosity and sufficient biomechanical support. The effect of the method used for the scaffolds’ fabrication, as well as the biochemical composition and biophysical properties of the scaffolds, including pore size, on driving stem cells towards the osteogenic lineage, have been extensively described in the literature [8,9]. In particular, interconnected porous structures with pore sizes ranging between 100 and 325 μm have been reported for various bone tissue engineering scaffolds as essential for cell growth and the exchange of nutrients [10,11,12]. The overall shape, porosity, pore size and distribution, along with surface topography and internal structure of the scaffold play a crucial role in regulating cell functions and bone ingrowth [13,14]. The choice of the materials and the fabrication method are critical factors in the development of scaffolds for bone tissue engineering applications with a significant impact on the scaffolds’ properties [15].

In recent years, biomimetic hybrid composites that combine the advanced mechanical characteristics of synthetic polymers with the bioactivities of naturally occurring biopolymers have gained great interest for bone regeneration applications [16]. Among synthetic polymers, polycaprolactone (PCL) has been widely used in the fabrication of bone tissue engineering scaffolds, despite its low degradation rate, due to its biocompatibility, biodegradability, relatively low immunogenicity, high mechanical strength and elasticity [17,18]. However, due to its highly hydrophobic nature, PCL lacks cell recognition signals. The absence of biological adhesion sites on the surface of PCL scaffolds hinders cell response, restricting its bone regeneration potential [19]. Nevertheless, the osteoinductivity of PCL scaffolds could be enhanced with the incorporation of natural polymers with osteoinductive ability into the PCL polymer matrix [20]. To improve the cellular behavior of PCL-based biomaterials, several attempts have been made, including blending with natural hydrophilic polymers, such as gelatin, collagen, and chitosan, incorporating a second component with significantly better biological properties, and modifying its surface [21,22,23,24,25,26,27].

Natural polymers represent an emerging class of materials in the biomedical sector due to their wide range of biological activities, as well as the high biocompatibility and biodegradability they exhibit [28]. Among them, marine polysaccharides are considered non-toxic, easy-to-access biopolymers with a diverse spectrum of bioactivities [29,30,31]. Their inherent biological properties, structural and chemical diversity, and resemblance to glycan constituents of the natural ECM renders them ideal biomaterials for the development of novel biosystems for tissue engineering and other biomedical applications [32,33,34,35]. Chitosan, for example, is one of the most widely known marine polymers finding applications in tissue engineering, not only as a scaffold component, but also as a therapeutic delivery platform for promoting in vitro and in vivo bone and cartilage repair [36,37].

Ulvan is a complex anionic sulfated polysaccharide, abundantly present in the cell walls of green seaweeds of the order Ulvales [38,39]. Exhibiting strong biological activities, such as antioxidant, antiviral, anticoagulant, antihyperlipidemic, immunomodulating and antitumor [40,41,42,43,44,45], ulvan has been gaining over the last years increased interest for the development of novel biomedical scaffolds [46,47,48]. Due to its chemical similarity to glycosaminoglycans, intrinsic cytocompatibility and gelling properties, ulvan has been utilized for the preparation of various hybrid scaffolds, polymeric nanofibers, 2D crosslinked membranes, multilayer nanofilms and polyelectrolyte complexes for an array of biomedical applications [38,49,50,51,52,53,54,55,56,57]. Over the last years, the osteoinductive potential of ulvan has attracted the attention of researchers for the development of scaffolds for osseous tissue regeneration [32,58,59,60].

In the framework of our ongoing research towards the development of ulvan-based biomaterials [52,53,54,55,60], we report herein the results of our investigations on the osteoinductive ability of ulvan, utilized in the preparation of a series of hybrid ulvan/PCL (UP) scaffolds. We hypothesized that the incorporation of ulvan into the PCL matrix could efficiently promote the attachment and proliferation of seeded cells, without negatively affecting the mechanical properties of the designed scaffolds. Moreover, we envisaged that the different incorporation methodology of ulvan into the PCL matrix and the presence of additional marine biopolymers could alter the mechanical and osteoinductive properties of the designed scaffolds. Therefore, a series of hybrid UP scaffolds, containing ulvan either alone or in blends with other previously reported as osteoinductive marine sulfated polysaccharides [33,61,62,63], namely κ-carrageenan and chondroitin sulfate, was prepared and physicochemically characterized. The impact of the preparation methodology and polysaccharide content on their morphology and their thermal, water uptake, porosity and mechanical properties was determined. Their bone tissue regeneration potential was investigated through the evaluation of cell adhesion, viability, and osteogenic differentiation of human adipose-derived mesenchymal stem cells (hADMSCs) seeded on the prepared UP scaffolds.

## 2. Results and Discussion

**Figure 1:**
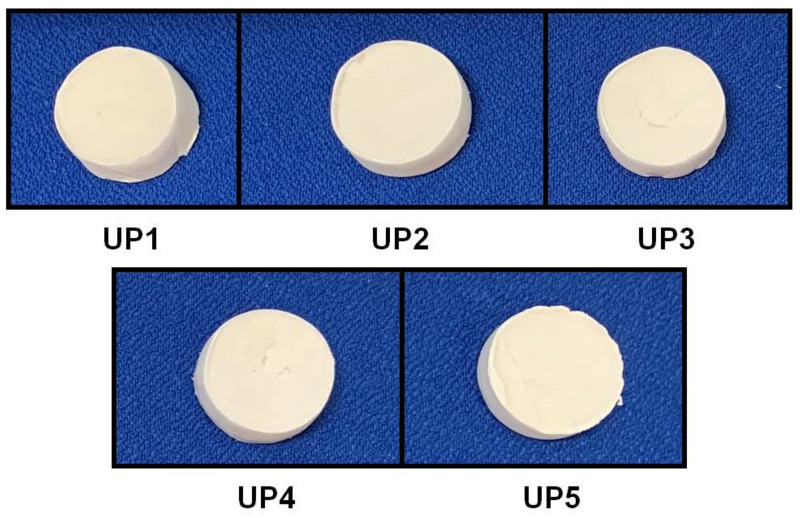
Images of the ulvan/polycaprolactone (UP) scaffolds.

All UP composites (Figure 1) were prepared as freeze-dried PCL-based scaffolds of a 1:10 (*w*/*w*) total-polysaccharide to PCL ratio. In order to investigate the impact of the preparation technique on the osteoinductive capacity of the fabricated scaffolds, ulvan and PCL were employed for the preparation of UP1 and UP5 following two different methodologies. Specifically, for the preparation of UP1 scaffold, ulvan was homogenously dispersed into a solution of PCL in benzene, whereas for the preparation of UP5 scaffold, ulvan was initially dissolved in distilled water and subsequently added to a benzene solution of PCL and rigorously mixed towards the formation of a benzene/water emulsion. In a further step and in order to investigate if the presence of additional marine-derived polysaccharides could enhance the osteoinductive properties of the UP scaffolds, ulvan in blends with κ-carrageenan or/and chondroitin sulfate was homogenously dispersed into a benzene PCL solution towards the preparation of UP2, UP3 and UP4 scaffolds.

**Figure 2:**
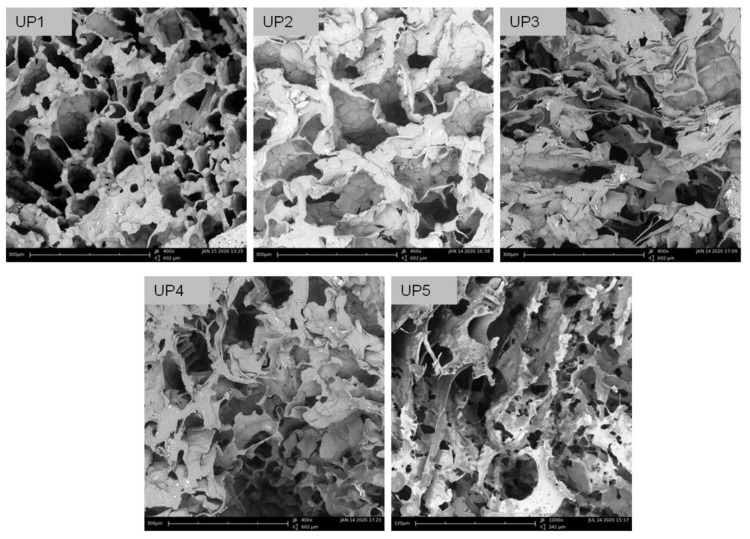
SEM images of the UP scaffolds.

The analysis of the images obtained using scanning electron microscopy (SEM) revealed the morphological characteristics of the UP scaffolds (Figure 2). All scaffolds demonstrated a macroporous network with interconnected pores and large surface areas suitable for cell adhesion and cell growth. Specifically, UP1, UP2, UP3 and UP4 exhibited a 3D porous structure organized in a coral reef-like morphology. UP1 scaffold demonstrated the most homogeneous pore anatomy compared to the others, while the different polysaccharide content in UP2, UP3 and UP4 did not significantly affect the morphological characteristics of the scaffolds. The higher amount of ulvan loaded in the UP1 scaffold most probably contributed to this network homogeneity due to its content in the more hydrophobic rhamnose. UP5 scaffold presented different structural characteristics, as a result of its preparation method (emulsion), exhibiting a sponge-like morphology with a lamellar-like porous architecture. In all cases, the interconnected networks lacked any specific structural motifs, since randomly oriented pores of different shapes and sizes were observed. Specifically, pore sizes ranged from 52 to 253 μm for UP1, 47 to 275 μm for UP2, 63 to 239 μm for UP3 and 54 to 189 μm for UP4, while UP5 revealed smaller pores with sizes ranging from 27 to 141 μm. Mean pore sizes were determined at 150, 142, 125, 121 and 62 μm for UP1, UP2, UP3, UP4 and UP5 scaffolds, respectively. Taking into account that pore sizes in the range of 50–300 μm are beneficial for tissue growth and nutrient delivery, all scaffolds exhibited pore sizes suitable for cell colonization and proliferation.

Porosity and density measurements of the prepared UP scaffolds are presented in Table 1. While UP1, UP2, UP3, and UP4 scaffolds exhibited slightly lower porosity values compared to that of UP5, all scaffolds exhibited high porosity ranging between 79% and 82%. Considering the fact that spongy bone porosity ranges from 30% to 90% [64], all fabricated scaffolds could meet the requirements for bone tissue engineering due to their interconnected and highly porous structure. Moreover, the UP scaffolds demonstrated densities, ranging between 0.181 g/cm^3^ and 0.202 g/cm^3^. The recorded densities of UP1, UP2, UP3, and UP4 were similar and slightly higher to that of the UP5 (0.181 g/cm^3^) and the PCL (0.187 g/cm^3^) scaffolds, indicating that the density of the scaffolds was independent of the polysaccharide content, as also evidenced by the evaluation of their mechanical properties.

**Figure 3:**
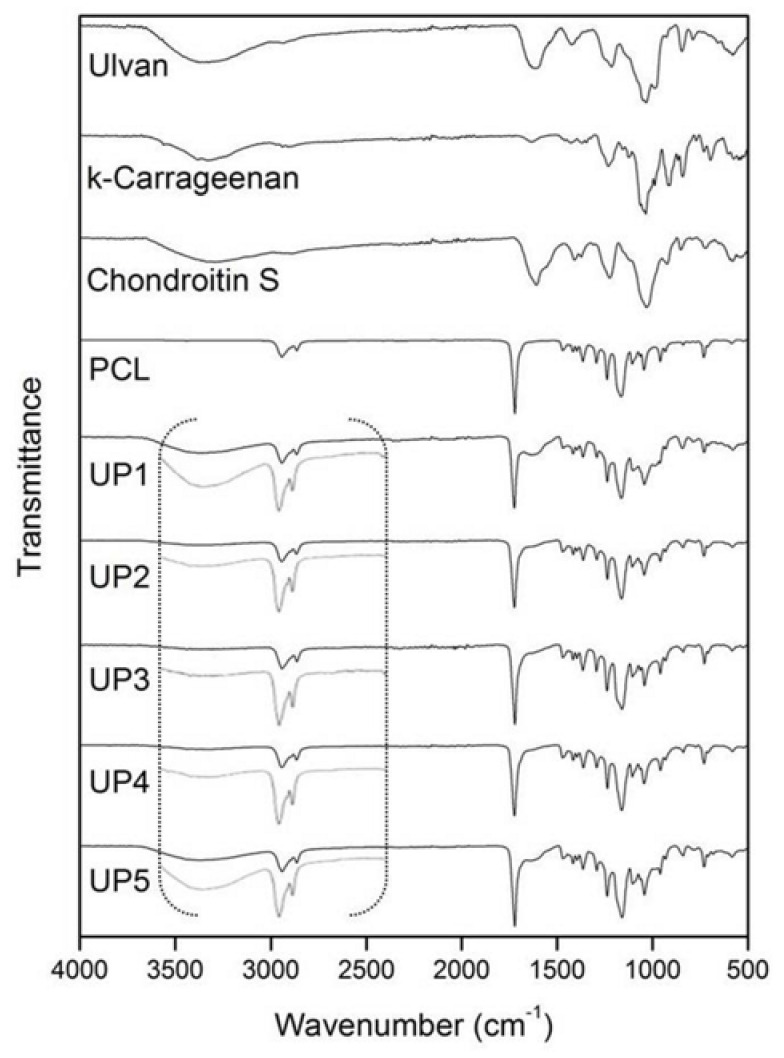
FTIR spectra of ulvan, κ-carrageenan, chondroitin sulfate, polycaprolactone (PCL) and UP scaffolds.

The FTIR spectrum of ulvan (Figure 3) exhibited the most characteristic absorption bands at 3367 cm^−1^, 1606 cm^−1^ and 1035 cm^−1^ assigned to the stretching vibrations of −OH, −C=O carboxylic groups and ether glycosidic linkage (C−O−C), respectively. The sulfate ester groups showed absorption bands at 1216 cm^−1^ and 847 cm^−1^. κ-Carrageenan exhibited –OH and glycosidic linkage (C−O−C) stretching vibrations at 3326 cm^−1^ and 1037 cm^−1^, respectively, while the absorption bands of sulfate esters were observed at 1231 cm^−1^ and 843 cm^−1^. For chondroitin sulfate, the characteristic peak at 3311 cm^−1^ was assigned to −OH and −NH stretching vibrations. The glycosidic linkage (C−O−C) absorption was evident at 1031 cm^−1^, while the stretching vibration at 1606 cm^−1^ was assigned to −C=O carboxylate and amide bonds. Sulfate esters showed absorption bands at 1224 cm^−1^ and 849 cm^−1^.

Due to the large amount of PCL dominating the scaffolds, all UP scaffolds exhibited similar spectra revealing mainly the characteristic absorption bands of PCL. The absorption bands at 2942 cm^−1^ and 2865 cm^−1^ were assigned to the −CH_2_ stretching vibration, the carbonyl −C=O stretching was observed at 1721 cm^−1^, while the C−O and C−C stretching were recorded at 1166 cm^−1^. The incorporation of the polysaccharides into the UP scaffolds was evident in their FTIR spectra by the characteristic absorption of the saccharide structures at approximately 3367 cm^−1^ attributed to the −OH stretching (Figure 3).

**Figure 4:**
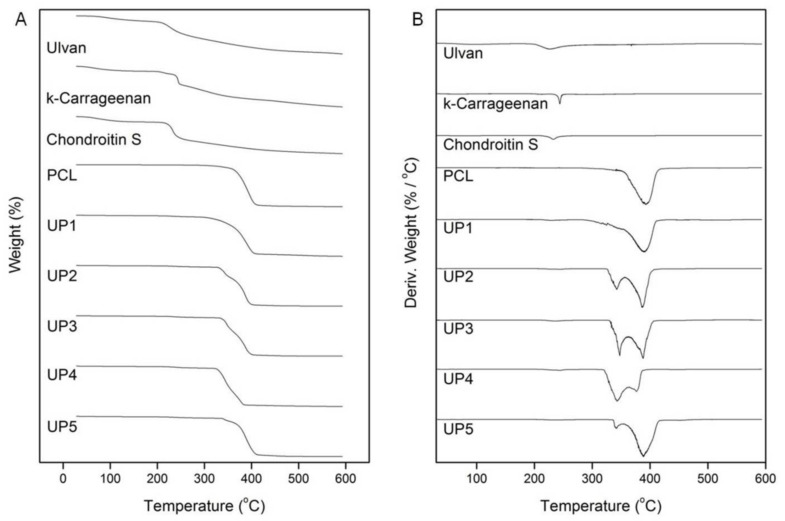
(**A**) TGA and (**B**) DTG thermograms of ulvan, κ-carrageenan, chondroitin sulfate, PCL and UP scaffolds.

The thermal stability of the UP scaffolds was investigated by TGA analysis (Figure 4). In the TGA thermograms, ulvan, κ-carrageenan and chondroitin sulfate showed a slight mass loss up to 205 °C, 201 °C and 214 °C, respectively, attributed to the volatilization of absorbed moisture and hydrogen-bound water. Above these temperatures, an increasing rate in their mass loss was observed up to their inflection points which were recorded at 227 °C, 241 °C and 232 °C, respectively, due to decomposition phenomena associated to dehydration of the sugars and cleavage of C−H and C−O−C glycoside bonds in the main polysaccharide chain. The thermal decomposition of PCL was initiated at 320 °C, followed by a fast mass loss from 350 °C up to its inflection point at 390 °C and its degradation was completed at around 410 °C. All UP scaffolds exhibited different thermogravimetric curves revealing the degradation phenomena of their components. For UP1, the initial decomposition temperature was recorded at 205 °C with a first slight mass loss up to 227 °C, attributed to the degradation of ulvan. The major decomposition initiated at 308 °C, was followed by a fast mass loss until the inflection point of PCL at 390 °C and completed at 406 °C. UP2 started to decompose at 205 °C. The inflection points of ulvan and κ-carrageenan were recorded at 227 °C and 245 °C, while the main decomposition occurred between 328 °C and 400 °C due to the thermal degradation of PCL. Similarly, UP3 started to decompose at 205 °C with a slight mass loss up to 232 °C due to the degradation of ulvan and chondroitin sulfate, while the main mass loss occurred due to the decomposition of PCL between 330 °C and 385 °C and completed at 400 °C. The thermogram of UP4, revealed a similar thermal degradation pattern, with an initial decomposition temperature at 205 °C, and a first mass loss until 241 °C, corresponding to the degradation of polysaccharides. The main decomposition of the scaffold occurred from 323 °C to 383 °C. UP5 started to decompose at 205 °C with a slight mass loss up to 227 °C due to the decomposition of ulvan, while the main decomposition occurred from 337 °C to 410 °C.

In the DTG thermograms of the UP scaffolds (Figure 4), the maximum decomposition for UP1, UP2, UP3 and UP5 was recorded at 390 °C, while for UP4 maximum mass loss was recorded at 342 °C. This mass loss at 342 °C was also present in the DTG thermograms of UP2, UP3 and UP5 with higher intensity in UP3, lower in UP2 and even lower in UP5, pointing to their lower thermal stability compared to UP1 scaffold. This difference in the thermal stability could be attributed to the different polysaccharide content in the case of UP2, UP3 and UP4 since synergistic thermal events rising from the degradation of the polysaccharide mixture most probably favor this fast mass loss rate at a lower temperature. In the case of UP5, this thermal instability could be due to the less uniform composition resulting from the different fabrication method of the scaffold.

**Figure 5:**
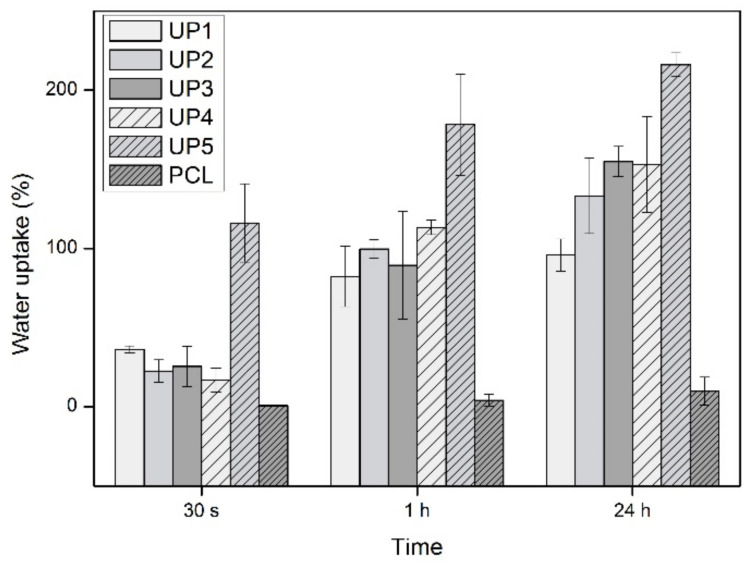
Water uptake ability (%) of PCL and UP scaffolds as a function of time (*n* = 3).

In the water uptake ability investigations, the fabricated scaffolds reached their water uptake maximum after a period of 24 h (Figure 5). Compared to the neat PCL, all UP scaffolds demonstrated significantly higher water uptake ability. At all time intervals, the PCL scaffold showed very low water absorption that was measured at 0.4% in the first 30 s, increased at 4% after 1 h and reached its maximum at 8% in 24 h. UP1, UP2, UP3 and UP4 scaffolds showed water absorption of 36%, 23%, 25% and 17% at 30 s that increased after 1 h to 82%, 100%, 89% and 113% and reached their maximum after 24 h at 95%, 133%, 154%, and 153%, respectively. The differences in the water absorption among these scaffolds could be attributed to the different polysaccharide compositions. However, UP5 demonstrated the highest water uptake values at all time intervals. This might be attributed to its different fabrication method that resulted in different pore structures. Its water uptake ability was recorded at 116% at 30 s, increased up to 178% in 1 h and reached its maximum at 216% after 24 h. It is obvious that the hydrophilic nature of the embedded polysaccharides into the PCL matrix improved the water uptake ability of the fabricated scaffolds. It is worth noting that the water uptake efficiency of the designed scaffolds is of high importance for cell adhesion and proliferation, potentially enhancing their bone tissue engineering function.

The results of the compressive modulus of elasticity are presented in Table 2. No statistically significant differences were observed among the different scaffolds, implying that differences in methodology and composition did not affect the modulus. The measured values for the UP scaffolds are lower than previously reported ones for bulk mechanical properties of PCL (ranging from 16 to 32 MPa), a finding which could be attributed to their extended porosity [65].

**Figure 6:**
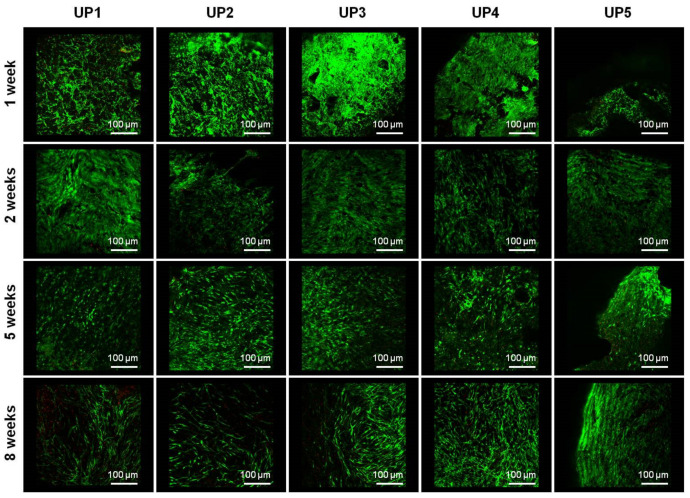
Confocal images of UP scaffolds seeded with human adipose-derived mesenchymal stem cells (hADMSCs), recorded at 4× magnification. Live/dead staining with calcein AM/ethidium homodimer shows cell viability of hADMSCs after 1 week culture in MSC expansion medium and 2, 5 and 8 weeks culture in osteogenic differentiation medium. For each scaffold, ten serial sections were taken with a step/section set at 50 μm in the Z direction. In total, an area of 500 μm in height was analyzed for each scaffold at each time point, so both cell attachment and penetration along the *z*-axis could be visualized.

Cell viability was determined by confocal microscopy. During the first week of expansion in the MSC medium, the cells seeded on the fabricated scaffolds showed enhanced spreading and viability with a very low number of dead cells observed, depicted as red dots in the fluorescent images (Figure 6). In all scaffolds, the cells remained viable during the cultivation period in osteogenic medium, showing elongated morphology and good spreading. In prolonged culture, no significant batches of dead cells were observed, since cells in all cases exhibited high viability. After 1 week of culture in MSC expansion medium or 2, 5 and 8 weeks of cultivation in osteogenic differentiation medium cell viability was determined from 97.5% to 99.2% for UP1, from 96.7% to 98.6% for UP2, from 94.1% to 98.9% for UP3, from 94.8% to 99.7% for UP4 and from 94.1% to 97.6% for UP5 scaffolds, respectively (Figure S1).

**Figure 7:**
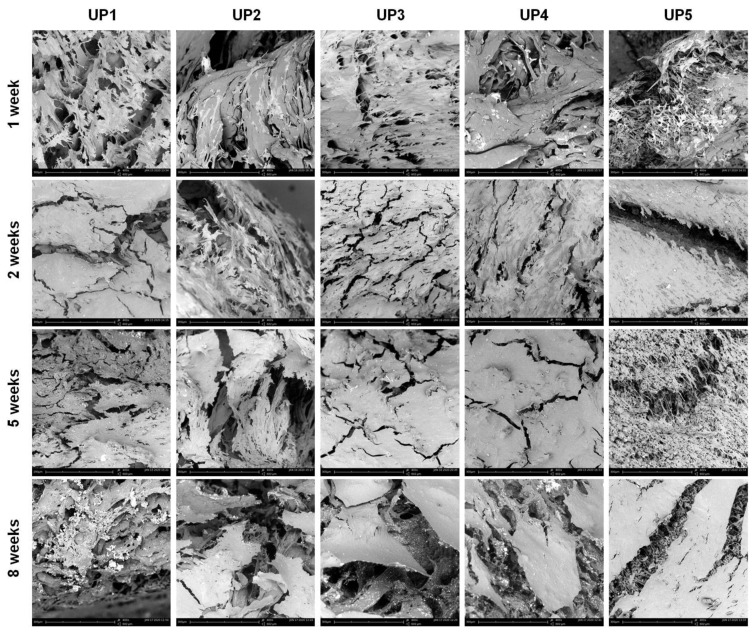
SEM images of UP scaffolds seeded with hADMSCs, depicting the adhesion and spreading of hADMSCs on the porous structure of the scaffolds after 1 week culture in MSC expansion medium and 2, 5 and 8 weeks culture in osteogenic differentiation medium.

Cell adhesion, morphology and osteogenic differentiation of cells were examined by scanning electron microscopy (Figure 7 and Figure S2). After 1 week of cultivation in MSC expansion medium, significant spreading of cells was observed on the surface of the scaffolds. This phenomenon was more evident in UP1, as well as in UP5, and to a lesser extent in UP2, UP3 and UP4 composites, as a higher number of cells seeded on the scaffold surface was observed. The cells seeded on the scaffolds formed bridges with neighboring cells, exhibiting a spike-like radiant morphology with filopodia and lamellipodia extensions, which is indicative of good cellular attachment and migration. After 2 weeks of cultivation in an osteogenic differentiation medium, morphological changes were observed as the cells started to show an elongated and flattened structure with short cytoplasmatic protrusions. The cells were found to be spread in wide areas, and colonize the scaffolds forming a cell layer, exhibiting enhanced cell growth and proliferation. After 5 weeks in the osteoinductive growth medium, cells occupied large areas of the scaffolds, forming an extended layer of ECM. After 8 weeks of cultivation, dense and continuous cell sheets were observed, covering almost the entire surface of the scaffolds. In general, the evaluation of the SEM images revealed a faster cell differentiation rate on the surfaces of UP1 and UP5 scaffolds in comparison to the other composites. Cross-sections of the UP scaffolds at different time points showed the penetration of the cells in their pores (Figure S3).

**Figure 8:**
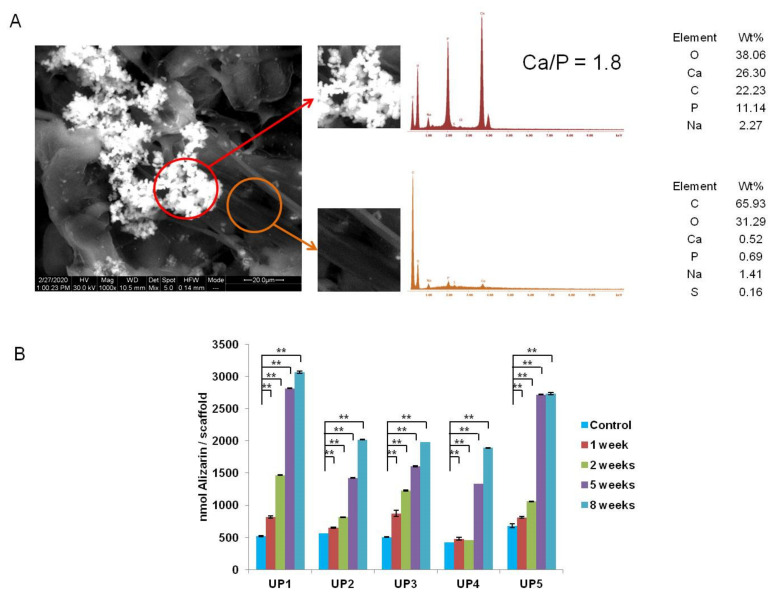
(**A**) EDS spectra of the mineralized nodules (red circle) and of the non-mineralized area (orange circle) of the UP1 scaffold. (**B**) Quantification of the matrix mineralization. Calcium incorporation in the ECM increases with cultivation time of hADMSCs seeded in UP scaffolds cultured in MSC expansion medium for 1 week and in osteogenic differentiation medium for 2, 5 and 8 weeks (*n* = 3). Asterisks indicate statistically significant differences compared to the control cultures at each time point (** *p* < 0.01).

During the 5th week, mineralized nodules were observed on the surfaces of the samples and by the 8th week, cell mineralization was clearly evident on the surface of all scaffolds. The amount of the particles rapidly increased and the mineralized nodules were found to be spread in wide areas of the scaffolds, while in some areas they were organized in dense grape-like structures. EDS analysis (Figure 8A) clearly showed the calcium and phosphorus composition of all mineralized particles, with an atomic Ca/P ratio of 1.8 which corresponds well to the Ca/P ratio of hydroxyapatite (1.67). The formation of mineralized particles on the surface of the scaffolds indicates the presence of an “apatite” layer that could serve as a precondition stage for induction of osteogenesis. Mineralization was more evident on the SEM images of UP1 and UP5, where more mineralized particles and denser particle structures were observed. This could be attributed to the higher amount of ulvan present in the UP1 and UP5 composites that may provide more favorable conditions for cell adhesion and spreading.

Alizarin red was used for the detection and quantification of the ECM mineralization of hADMSCs seeded in UP scaffolds after 2, 5 and 8 weeks of osteoinductive cultivation. As the calcification of the matrix is related to the differentiation level of the cell culture, mineralization is considered a marker of osteoblast differentiation. Figure 8B shows a strong increase in mineralization of cells that were induced to osteogenic differentiation throughout the culture period compared to unstimulated MSCs. The measured values were statistically higher compared to the baseline expression of the control UP scaffolds, at all time points (*p* < 0.01 in all cases) (Figure 8B). No calcium precipitation was observed on the cell-free scaffolds used as controls, providing evidence that ECM mineralization occurred as a result of a biological process by the cells. UP1 showed the highest alizarin expression after 2 weeks of cultivation compared to the other UP scaffolds (UP1: 1468 ± 7.56-fold, UP2: 813 ± 4.8-fold, UP3: 1227 ± 10.34-fold, UP4: 456 ± 3.97-fold, and UP5: 1057 ± 4.5-fold). In addition, the highest calcium incorporation was observed in UP1 and UP5 scaffolds, with the most significant increase at 5 weeks (UP1: 2818 ± 5.65-fold and UP5: 2721 ± 7.7-fold), after which alizarin expression appeared to have increased slightly at 8 weeks cultivation in osteogenic medium (UP1: 3066 ± 17.53-fold and UP5: 2739 ± 17.46-fold).

**Figure 9:**
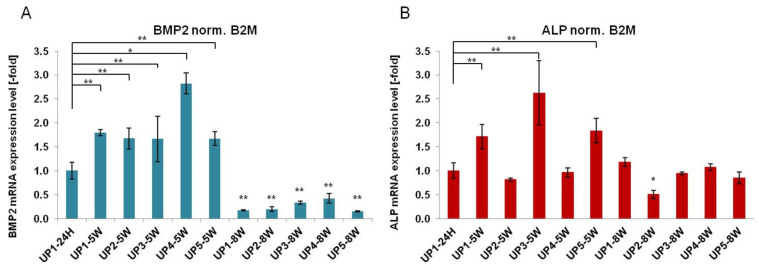
Gene expression of hADMSCs seeded in UP scaffolds. RT-PCR analysis of (**A**) bone morphogenetic protein-2 (BMP2) and (**B**) alkaline phosphatase (ALP) expression following culture in UP scaffolds for 5 and 8 weeks (*n* = 3). Asterisks indicate statistically significant differences compared to the control cultures at each time point (* *p* < 0.05, ** *p* < 0.01).

The promotion of osteogenesis in MSCs is dependent on a temporal pattern gene expression during the three major phases of osteogenesis, such as proliferation, matrix maturation and mineralization [66]. The osteogenic differentiation of hADMSCs in UP scaffolds was confirmed by RT-PCR analysis where the expression of two osteo-specific genes, such as bone morphogenetic protein-2 (BMP2) and alkaline phosphatase (ALP), was examined (Figure 9). Both BMP2 and ALP are among the most widely used osteogenic markers, mainly involved in bone mineralization [67,68]. Although, in general, the differentiation process starts earlier (after 1–2 weeks) [69], in the case of the hADMSC cells used in this study, preliminary experiments showed that the process started quite later and therefore measurements were performed at later time points (5 and 8 weeks).

Analysis of the expression levels of BMP2 showed that cells grown on all UP scaffolds exhibited upregulated expression compared to hADMSCs after 5 weeks of cultivation in osteogenic differentiation medium (UP1: 1.8 ± 0.06-fold, UP2: 1.67 ± 0.22-fold, UP3: 1.66 ± 0.47-fold, UP4: 2.82 ± 0.26-fold, and UP5: 1.67 ± 0.14-fold), with values statistically significantly higher compared to the baseline expression of UP1–24 h. At 8 weeks, BMP2 expression was statistically downregulated compared to the baseline expression of UP1–24 h (Figure 9A). In addition, the expression of ALP was significantly upregulated in UP1, UP3, and UP5 scaffolds seeded with hADMSCs after 5 weeks in culture, although to a lesser extent compared to BMP2 expression (UP1: 1.71 ± 0.26-fold, UP3: 2.62 ± 0.67-fold, and UP5: 1.84 ± 0.25-fold), with values statistically higher compared to the baseline expression of UP1–24 h (Figure 9B). This can be attributed to the fact that the cells are in the third phase of the differentiation program (the mineralization of the ECM), where ALP gene expression is downregulated. Osteo-specific gene expression confirmed that UP scaffolds can mediate osteogenesis differentiation of hADMSCs cultured in osteogenic media, suggesting that ulvan-based biomaterials possess suitable properties for bone tissue engineering applications.

The peak expression of alizarin in hADMSCs cultured in UP1 and UP5 at 5 weeks of differentiation can be correlated to the expression of ALP, an osteogenic marker that is known to accompany and influence the osteogenic differentiation. ALP levels gradually increased by day 14, after which the expression is decreased in both 5 and 8 weeks of osteogenic differentiation, suggesting an early calcium accumulation to the matrix and a limited augmentation of mineralization during later stages of differentiation. Since in the early stage of osteogenic differentiation ALP activity is upregulated [70,71], while in the late stage mineralization is involved [72,73], we believe that ALP expression during the early stage of osteogenesis acts as an upstream regulator required for enhanced mineralization in the final differentiation step in osteogenesis. Furthermore, UP2, UP3 and UP4 scaffolds are capable of osteogenic differentiation but seem to induce mineralization to a lower degree during the whole cultivation time compared to UP1 and UP5. This may be due to the endogenous difference in the osteogenic differentiation capacity between UP scaffolds. It is known that the interactions between cells and scaffolds can influence cytoskeletal organization which in turn can affect cell differentiation and expression of ECM. Thus, the different proportions of ulvan present in each scaffold can affect mineralization efficiency.

## 3. Materials and Methods

### 3.1. Materials

Polycaprolactone (PCL, MW 70,000–90,000), κ-carrageenan (MW > 950 kDa; 22.1% sulfate; rotational viscosity 14.5 mPa·s), chondroitin sulfate (sodium salt from shark cartilage; 6-sulfate:4-sulfate 1.64:1, sulfur 6.1%, sodium 7.0%) and benzene were obtained from Sigma-Aldrich (Darmstadt, Germany). All chemicals were of reagent grade and used without further purification. Biomass of the green alga *Ulva rigida* were collected from Chalkida bay, Greece, thoroughly cleaned from epiphytes, rinsed with seawater and fresh water, and air-dried. For the isolation of ulvan, 0.25 kg of the air-dried alga, grounded to small pieces, was macerated in 5 L of distilled water and heated in an autoclave for 20 min at 121 °C. After filtration of the hot aqueous solution through cotton cloth, the filtrate was allowed to cool at room temperature. Ethanol (96% *v*/*v*, 20 L) was added to the filtrate and the suspension was left overnight at 4 °C. The resulting precipitate was filtered through cotton cloth, washed thoroughly with ethanol, sonicated for 1 h in an ultrasonic bath, vacuum-filtered, and lyophilized to afford ulvan as an off-white powder, which was milled prior to use. Characterization of ulvan (MW distribution centered at approx. 1200 kDa; 48.1% sulfate, 40.3% carbohydrates; among carbohydrates rhamnose and uronic acids represented 25.2% and 18.1%, respectively) was performed as previously described [74].

### 3.2. Preparation of Ulvan/PCL Hybrid Scaffolds

All UP scaffolds were prepared by freeze-drying polymer solutions composed of the polysaccharides and PCL at a 1:10 (*w*/*w*) total polysaccharide to PCL ratio (Table 3). UP1, UP2, UP3 and UP4 scaffolds were prepared by dispersing the polysaccharides into a polymer solution of PCL in benzene. Specifically, PCL was dissolved in benzene at a 15% *w*/*v* concentration. Subsequently, ulvan either alone or in blends with κ-carrageenan or/and chondroitin sulfate was added to each PCL solution at 1.5% *w*/*v* (total polysaccharide) concentration. For the preparation of UP1 scaffold, pure ulvan was dispersed into the PCL solution. For the preparation of UP2, ulvan and κ-carrageenan at a 7:3 (*w*/*w*) ratio were added into the PCL solution. For the preparation of UP3, ulvan and chondroitin sulfate at a 7:3 (*w*/*w*) ratio were dispersed into the PCL solution, while for the preparation of UP4, ulvan, κ-carrageenan and chondroitin sulfate at a 2:1:1 (*w*/*w*) ratio were added into the PCL solution. For the preparation of UP5 scaffold, PCL in benzene at 15% *w*/*v* concentration and ulvan in water at 1.5% *w*/*v* concentration were blended in a 3:1 ratio to afford an emulsion. Control scaffolds were obtained by dissolving pure PCL in benzene at 15% *w*/*v* concentration. All polymer solutions were prepared at room temperature under stirring for 24 h to ensure homogeneity. Subsequently, they were placed in appropriate cylindrical molds (20 × 40 mm size), frozen at −20 °C overnight, and finally lyophilized at −110 °C for 24 h to afford the UP freeze-dried scaffolds. All subsequent measurements, apart from the determination of porosity and density and mechanical compression, were performed on scaffolds prepared in a single batch. Nonetheless, all measurements were performed with the same lot of materials.

### 3.3. Scanning Electron Microscopy and EDS Analysis

A PhenomWorld desktop scanning electron microscope (SEM, Thermo Fischer Scientific, Waltham, MA, USA) with a tungsten filament (10 kV) and charge reduction sample holder was used for the morphological characterization of the UP scaffolds. Additionally, the morphology of the UP scaffolds seeded with hADMSCs after 1 week proliferation in mesenchymal stem cells (MSC) expansion medium and 2, 5 and 8 weeks culture in osteogenic differentiation medium was also observed with SEM. Scaffolds were fixed with 3% *v*/*v* glutaraldehyde, rinsed, and then dehydrated in increasing concentrations (30–100% *v*/*v*) of ethanol in water. The samples were dried and observed without conductive coating. Elemental analysis was performed on a FEI QUANTA 200 (FEI Technologies Inc., Hillsboro, OR, USA) scanning electron microscope equipped with an energy dispersive X-ray analysis (EDS) detector.

### 3.4. FTIR Spectroscopy

Fourier transform infrared (FTIR) spectra of the UP scaffolds were measured on a FTIR Bruker Alpha II (Billerica, MA, USA) using the attenuated total reflection method.

### 3.5. Thermogravimetric Analysis

Thermogravimetric analysis was performed using a TA Thermogravimetric Analyzer (TGA 55, TA instruments, New Castle, DE, USA) under a nitrogen flow of 25 mL/min at a heating rate of 10 °C/min from 40–600 °C. Sample temperature, sample weight and heat flow were recorded continuously.

### 3.6. Determination of Porosity and Density

The porosity of the UP scaffolds was determined using a method based on the relative density of the prepared UP scaffolds [75]. The volume of the scaffolds was calculated using vernier callipers to measure sample dimensions, while a mass balance was used to measure the mass of the scaffold.

The relative density of the scaffolds was determined and the porosity of the scaffolds was calculated using the following equation:

$$Porosity\left(\%\right)=1-\left(\frac{P\;scaffold}{P\;material}\right)\times 100$$

where *P scaffold* is the apparent density of the scaffolds measured by dividing the mass by the volume of the scaffold and *P material* is the density of the material from which the scaffold is fabricated. The mean value of six cylindrical samples (diameter = 10 mm and height = 20 mm) was taken as the relative porosity percentage of each scaffold.

### 3.7. Determination of Water Uptake Ability

The water uptake ability of the UP scaffolds was evaluated by immersion of pre-weighted samples in distilled water (1% *w*/*v*) at RT for different time intervals (30 s, 1 h and 24 h). After each time point, each wet sample was placed on a blotting paper for 1 min to remove the excess surface water. Subsequently, the wet sample was weighted and the water uptake degree was calculated using the following equation:

$$\mathrm{Water}\;\mathrm{Uptake}\left(\%\right)=[\left(Ww-Wd\right)/Wd]\times 100$$

where *Ww* is the weight of the wet sample and *Wd* is the initial weight of the dry sample. Measurements were performed in triplicate.

### 3.8. Determination of Mechanical Compression

Six cylindrical samples (diameter = 10 mm and height = 20 mm) for each scaffold were prepared and tested employing a universal testing instrument (Tensometer 10, Monsanto, Swindon, UK). The samples were loaded under compression at a crosshead speed of 25 mm/min at ambient temperature and the loading surfaces of the metallic compressive plates were covered with a thin layer of lubricant to eliminate friction. Stress–strain diagrams were recorded for all samples and the modulus of elasticity in compression was estimated by the slope of the initial linear part of the curve.

### 3.9. Statistical Analysis

Results are expressed as mean values ± standard deviation. Statistical comparison was performed using analysis of variance (ANOVA) single factor test and differences were considered statistically significant when *p* < 0.05. For BMP2, ALP and alizarin measurements, results are expressed as mean values ± standard deviation of 3 replicates repeated 3 times. Statistical comparison was performed using two-way ANOVA and Tukey’s post-hoc test was used for multiple comparisons between groups. All analyses were performed using Prism 6.0 Software (GraphPad, CA, USA) at two levels of statistical significance (* *p* < 0.05 and ** *p* < 0.01).

### 3.10. Isolation, Culture and Characterization of hADMSCs

The collection of human adipose tissue was performed in accordance with the local Ethical Commission (Papageorgiou Hospital, Review Board-approved protocols 263-7/12/2016) and after informed consent of all donors. hADMSCs were isolated from adipose tissue by enzymatic digestion in the cGMP facility as previously described and expanded in MSC medium consisting of a-MEM, 15% FBS, 2 mM glutamine, 0.1 mM *L*-ascorbic acid phosphate, 100 U/mL penicillin and 100 mg/mL streptomycin. Culture medium was replaced twice a week and cells were amplified up to P3. Flow cytometry was performed using Guava^®^ easyCyte 8HT (Merck-Millipore, Darmstadt, Germany) to analyze and characterize the isolated MSCs for cell surface and intracellular antigen expression. Cells expressed CD90 and CD73 (positive markers), whereas CD45 was not expressed, fulfilling the minimum criteria of the International Society for Cellular Therapy for defining multipotent MSC. Unstained cells were used as a control to set the gates and analysis was performed as previously described [25].

### 3.11. Cell Seeding on Scaffolds and Osteogenic Differentiation of hADMSCs

Scaffolds were cut into discs of 6 mm diameter using a skin biopsy punch under aseptic conditions, placed into 24-well plates and immersed in growth medium for 24 h before seeding to allow protein absorption on them. A cell suspension of 1 × 10^5^ cells dispersed in 20 μL of growth medium was seeded onto the scaffolds followed by 1 h incubation at 37 °C and 5% CO_2_ to allow cells to adhere. Subsequently, growth medium (1.5 mL/scaffold) was gently added [76]. After 1 week of culture in MSC expansion medium, the cell-seeded scaffolds were induced to differentiate into osteocytes using osteogenic medium (a-MEM supplemented with 15% FBS, 2 mM glutamine, 0.1 mM l-ascorbic acid phosphate, 100 U/mL penicillin, 100 mg/mL streptomycin, 10 nM dexamethasone, 1.8 mM KH_2_PO_4_ and 5 mM glycerolphosphate) and cultured for 2, 5 and 8 weeks (the medium was replaced twice per week).

### 3.12. Confocal Microscopy

Cell viability was monitored using the Viability/Cytotoxicity Assay Kit for Animal Live and Dead Cells (#3000, Biotium, CA, USA), according to the manufacturer’s instructions. Scaffolds seeded with hADMSCs were evaluated for cell viability and colonization after 1 week of proliferation in MSC expansion medium. In addition, visualization of the live cells attached to the UP scaffolds was examined after 2, 5 and 8 weeks culture in osteogenic differentiation medium. The constructs were captured as z-stack images using the EZ—C1 3.20 software. For live/dead staining, hADMSCs/scaffold constructs were doubly stained with calcein AM and ethidium homodimer, staining living and dead cells, respectively. For each scaffold, ten serial sections were taken with a step/section set at 50 μm in the Z direction. In total, an area of 500 μm in height was analyzed for each scaffold at each time point, so both cell attachment and penetration along the *z*-axis could be visualized. Fluorescence observations were performed by a confocal upright fluorescence microscope (Nikon D-Eclipse 80i C1). Quantification of fluorescence intensity was determined using Image J software (NIH, Bethesda, MD, USA). Corrected total cell fluorescence (CTCF) was calculated using the formula CTCF = Integrated Density − (Area of selected cell × Mean fluorescence of background readings) [77].

### 3.13. RNA Extraction and RT-PCR

Quantitative real-time reverse transcription polymerase chain reaction (qRT-PCR) was used to examine the osteogenic differentiation potential of UP scaffolds seeded with hADMSCs cultured in the presence of osteogenic medium. More specifically, total RNA was extracted from the constructs after 5 and 8 weeks in osteogenic medium using Nucleo-ZOL (Macherey Nagel, Düren, Germany), according to the manufacturer’s instructions. RNA purity and concentration were measured using a NanoDrop spectrophotometer (Epock, Biotek, Biotek instruments, Inc., Winooski, VT, USA). RNA samples were then reverse-transcribed using a superscript first-strand synthesis kit (Takara Bio USA, Inc., Mountain View, CA, USA), according to the manufacturer’s instructions. Reactions were performed using SYBR-Select PCR Master Mix (Applied Biosystems, Foster City, CA, USA) in a Step One Plus thermal cycler (Applied Biosystems) as previously described [76]. Primers were designed using the PRIMER BLAST for the following osteogenic genes: *BMP2*, F: GGAACGGACATTCGGTCCTT, R: AGTCCGTCTAAGAAGCACGC, *ALP* F: CCGTGGCAACTCTATCTTTGG R: CAGGCCCATTGCCATACAG. The results were adjusted by amplification efficiency (LinRegPCR) and normalized against the housekeeping gene beta-2-microglobulin (B2M) F: TGTCTTTCAGCAAGGACTGGT R: ACATGTCTCGATCCCACTTAAC, found to remain stable during differentiation processes of cells.

### 3.14. Alizarin Red Mineralization Assay

Osteogenic differentiation was assessed by alizarin red S staining. Briefly, seeded and cell-free (control) scaffolds were washed with PBS and fixed with 10% neutral buffered formalin (NBF) for 30 min at RT followed by 0.5% alizarin red (pH 4.2) staining for 60 min at RT. Afterwards, cells were washed three times with deionized water (dH_2_O) to reduce nonspecific staining. Subsequently, bound alizarin was unhinged by adding 10% cetylpyridinium chloride per scaffold and incubated for 1 h at RT. The concentration of the dye in each sample was determined by photometric absorption reading using a NanoDrop spectrophotometer on the basis of a standard curve with alizarin [μM]. Aliquots (200 μL) were transferred in a 96-well plate and the optical density (OD) of the solution was measured at 562 nm. For each measurement, the mean value was calculated by averaging the 3 single values of each group. For each sample, the amount of bound alizarin was determined as nmol alizarin/scaffold.

## 4. Conclusions

In summary, the osteoinductive ability of ulvan incorporated in porous freeze-dried PCL-based scaffolds was investigated. A series of hybrid UP scaffolds containing ulvan either alone or in blends with κ-carrageenan or/and chondroitin sulfate were prepared and the impact of the preparation methodology and polysaccharide content towards their bioactivity, physicochemical and mechanical properties was evaluated. All fabricated scaffolds containing 10% (*w*/*w*) polysaccharides of marine origin exhibited desirable characteristics for bone tissue engineering applications. They revealed uniform cell spread and adhesion, as well as high viability at all time intervals (1, 2, 5 and 8 weeks). The addition of κ-carrageenan and chondroitin sulfate did not seem to enhance the osteoinductivity of the scaffolds or alter their mechanical properties. UP1 scaffold exhibited overall the best characteristics, since it induced cell differentiation and mineralization only after 2 weeks of cultivation. Nevertheless, UP5 also reached maximum mineralization efficiency, equal to that of UP1, after 5 weeks of cultivation, evidencing the prime importance of the polysaccharidic component of the scaffold instead of the scaffold’s morphology.

The main scope of our study was to synthesize and characterize PCL/ulvan-based hybrid scaffolds and to provide a preliminary biological evaluation of the maintenance of cell attachment, spreading in the scaffolds and viability, as well as of the capacity to induce osteogenic differentiation and biomineralization. Further experiments to evaluate late differentiation markers for osteoblasts differentiation, such as osteopontin, osteocalcin, and the transcription factor RunX2, could be useful and the results should be compared to those obtained for the early differentiation markers tested for bone formation in this work, as well as to results pertaining to the evaluation of a PCL-alone scaffold prepared through the same methodology.

Even though ulvan is a sulfated polysaccharide with immunomodulatory properties, its low content (7–10% *w*/*w*) in the prepared UP scaffolds is not expected to be prohibitive for such applications. Nevertheless, in order to support the efficacy of the UP hybrid scaffolds in bone tissue regeneration, it would be necessary to evaluate their overall performance in vivo.

In conclusion, all scaffolds were found to promote the attachment and viability of seeded cells; however, UP1 and UP5 scaffolds, incorporating only ulvan in their polymer matrix, exhibited a faster differentiation rate, confirming the osteoinductive potential of ulvan towards the development of biomedical scaffolds for bone tissue regeneration applications.

## Figures and Tables

**Table 1:** Mean values and standard deviations of porosity and density of UP scaffolds (*n* = 6).

Scaffold	Porosity (%)	Density (g/cm^3^)
UP1	79.9 ± 0.4	0.196 ± 0.003
UP2	80.5 ± 0.3	0.195 ± 0.003
UP3	80.3 ± 0.3	0.197 ± 0.003
UP4	80.3 ± 0.4	0.202 ± 0.004
UP5	81.4 ± 0.2	0.181 ± 0.002
PCL	83.6 ± 0.4	0.187 ± 0.005

**Table 2:** Compressive modulus of elasticity of PCL and UP scaffolds (*n* = 6).

Scaffold	Modulus of Elasticity (MPa)
UP1	4.9 ± 2.1
UP2	3.8 ± 1.4
UP3	4.3 ± 0.7
UP4	4.1 ± 1.7
UP5	1.6 ± 0.1
PCL	3.7 ± 1.4

**Table 3:** Composition (*w*/*w*) of UP scaffolds.

Scaffold	PCL	Ulvan	κ-Carrageenan	Chondroitin Sulfate
UP1	100	10	0	0
UP2	100	7	3	0
UP3	100	7	0	3
UP4	100	5	2.5	2.5
UP5	100	10	0	0

## Data Availability

The data presented in this study are available in the present manuscript and respective Supplementary materials.

## Supplementary Materials

Supplementary materials are available online at https://www.mdpi.com/1422-0067/22/6/3086/s1.
