# Supplementary material for: The Marine Polysaccharide Ulvan Confers Potent Osteoinductive Capacity to PCL-Based Scaffolds for Bone Tissue Engineering Applications
Source: Int J Mol Sci. 2021 Mar 17;22(6):3086. doi: 10.3390/ijms22063086 (PMC8002638; doi:10.3390/ijms22063086)
Supplement: Supplementary file 1 [file ijms-22-03086-s001.pdf]

Supplementary Materials

The Marine Polysaccharide Ulvan Confers Potent Osteoinductive Capacity to PCL-based Scaffolds for Bone Tissue Engineering Applications

Stefanos Kikionis [1], Efstathia Ioannou [1], Eleni Aggelidou [2,3], Leto-Aikaterini Tziveleka [1], Efterpi Demiri [4], Athina Bakopoulou [2,5], Spiros Zinelis [6], Aristeidis Kritis [2,3] and Vassilios Roussis [1,*]

[1] Section of Pharmacognosy and Chemistry of Natural Products, Department of Pharmacy, National and Kapodistrian University of Athens, Panepistimiopolis Zografou, Athens 15771, Greece; skikionis@pharm.uoa.gr (S.K); eioannou@pharm.uoa.gr (E.I.); ltziveleka@pharm.uoa.gr (L-A.T.)

[2] cGMP Regenerative Medicine Facility, Department of Physiology and Pharmacology, School of Medicine, Faculty of Health Sciences, Aristotle University of Thessaloniki, Thessaloniki 54124, Greece; angelide@auth.gr (E.A.); abakopoulou@dent.auth.gr (A.B.); kritis@auth.gr (A.K.)

[3] Department of Physiology and Pharmacology, School of Medicine, Faculty of Health Sciences, Aristotle University of Thessaloniki, Thessaloniki 54124, Greece

[4] Department of Plastic Surgery, School of Medicine, Faculty of Health Sciences, Papageorgiou Hospital, Aristotle University of Thessaloniki, Thessaloniki 54124, Greece; demirie@auth.gr (E.D.)

[5] Laboratory of Prosthodontics, School of Dentistry, Aristotle University of Thessaloniki, Thessaloniki 54124, Greece

[6] Department of Biomaterials, School of Dentistry, National and Kapodistrian University of Athens, Athens 11527, Greece; szinelis@dent.uoa.gr (S.Z.)

[*] Correspondence: roussis@pharm.uoa.gr; Tel.: +30 210 727 4592

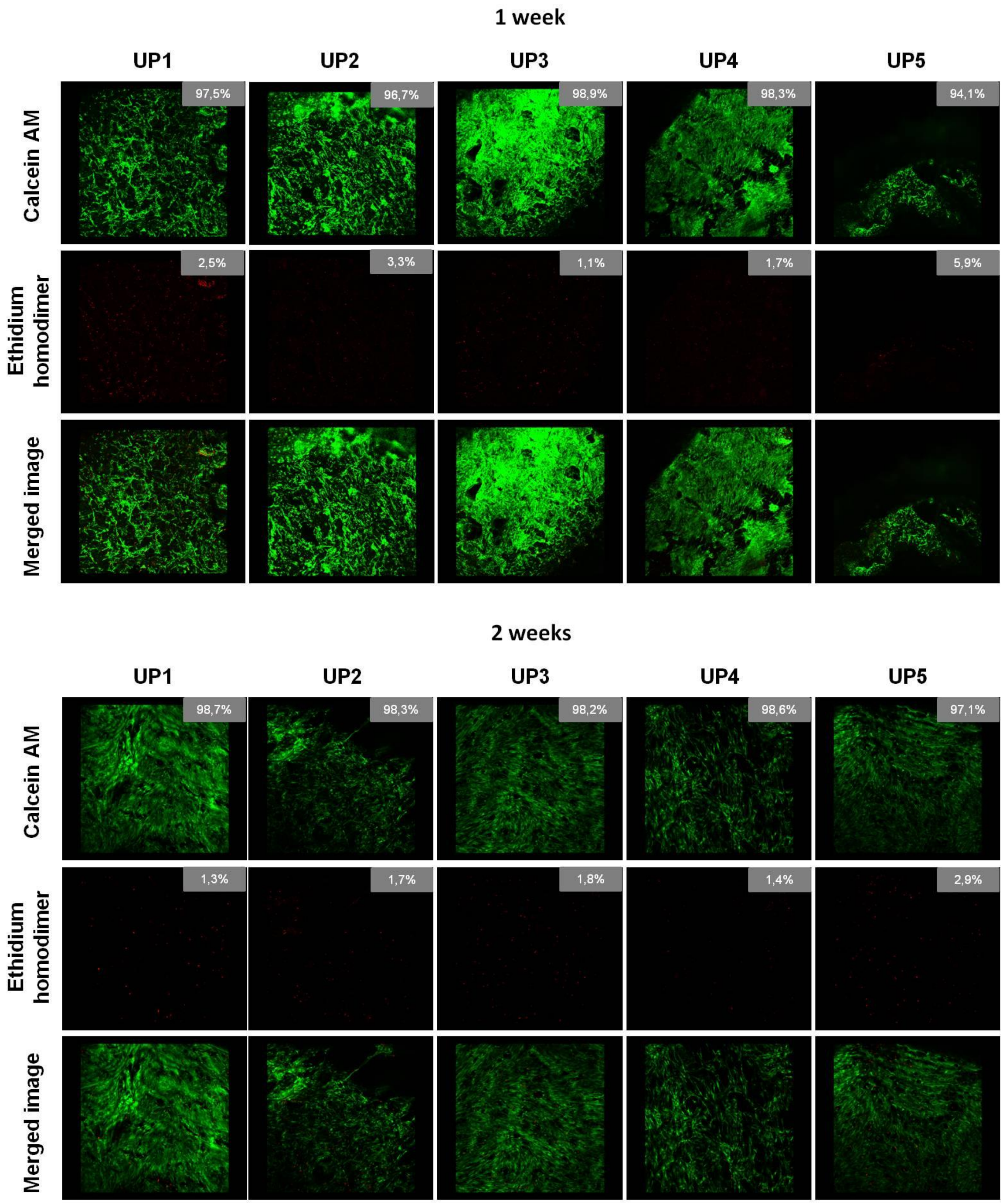
1 week
UP1
UP2
UP3
UP4
UP5
Calcein AM
97,5%
96,7%
98,9%
98,3%
94,1%
Ethidium homodimer
2,5%
3,3%
1,1%
1,7%
5,9%
Merged image
2 weeks
UP1
UP2
UP3
UP4
UP5
Calcein AM
98,7%
98,3%
98,2%
98,6%
97,1%
Ethidium homodimer
1,3%
1,7%
1,8%
1,4%
2,9%
Merged image

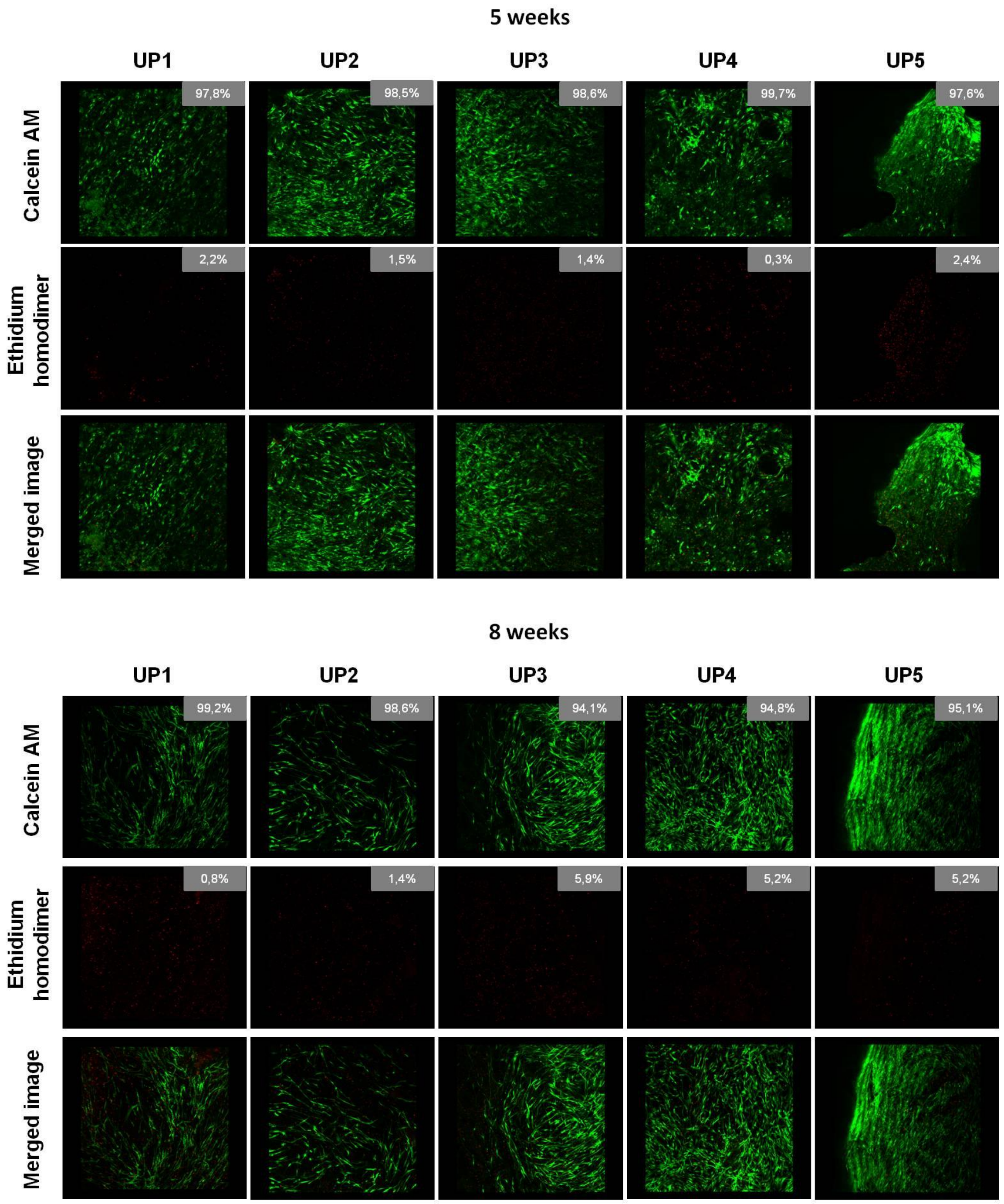

Figure S1. Confocal images of UP scaffolds seeded with hADMSCs. Live/dead staining with calceinAM/ethidium homodimer showing cell viability of hADMSCs after 1 week culture in MSC expansion medium and 2, 5 and 8 weeks culture in osteogenic differentiation medium. The relative cell viability or mortality (%) are denoted in the upper right part of the images.

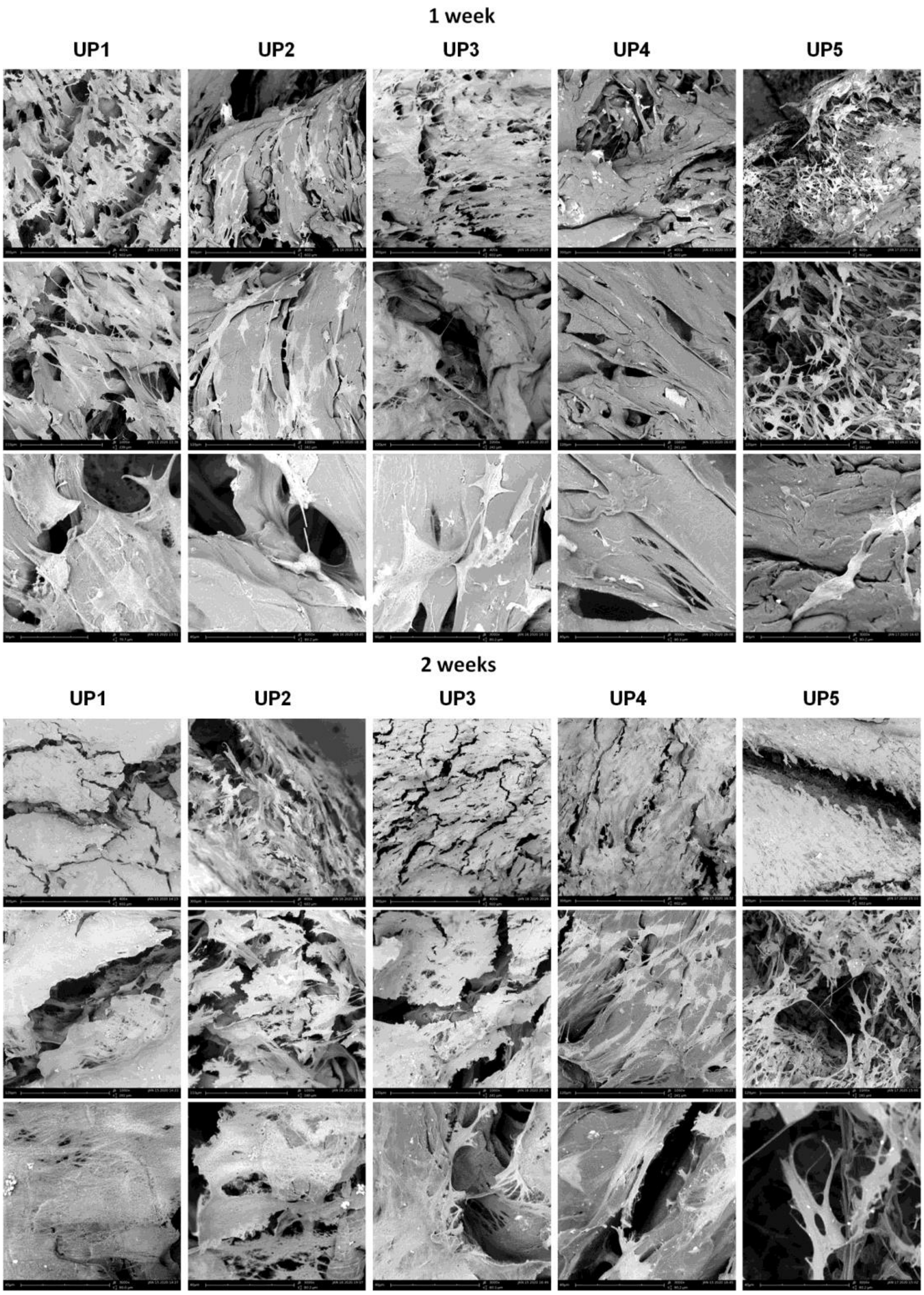
1 week
UP1
UP2
UP3
UP4
UP5
2 weeks
UP1
UP2
UP3
UP4
UP5

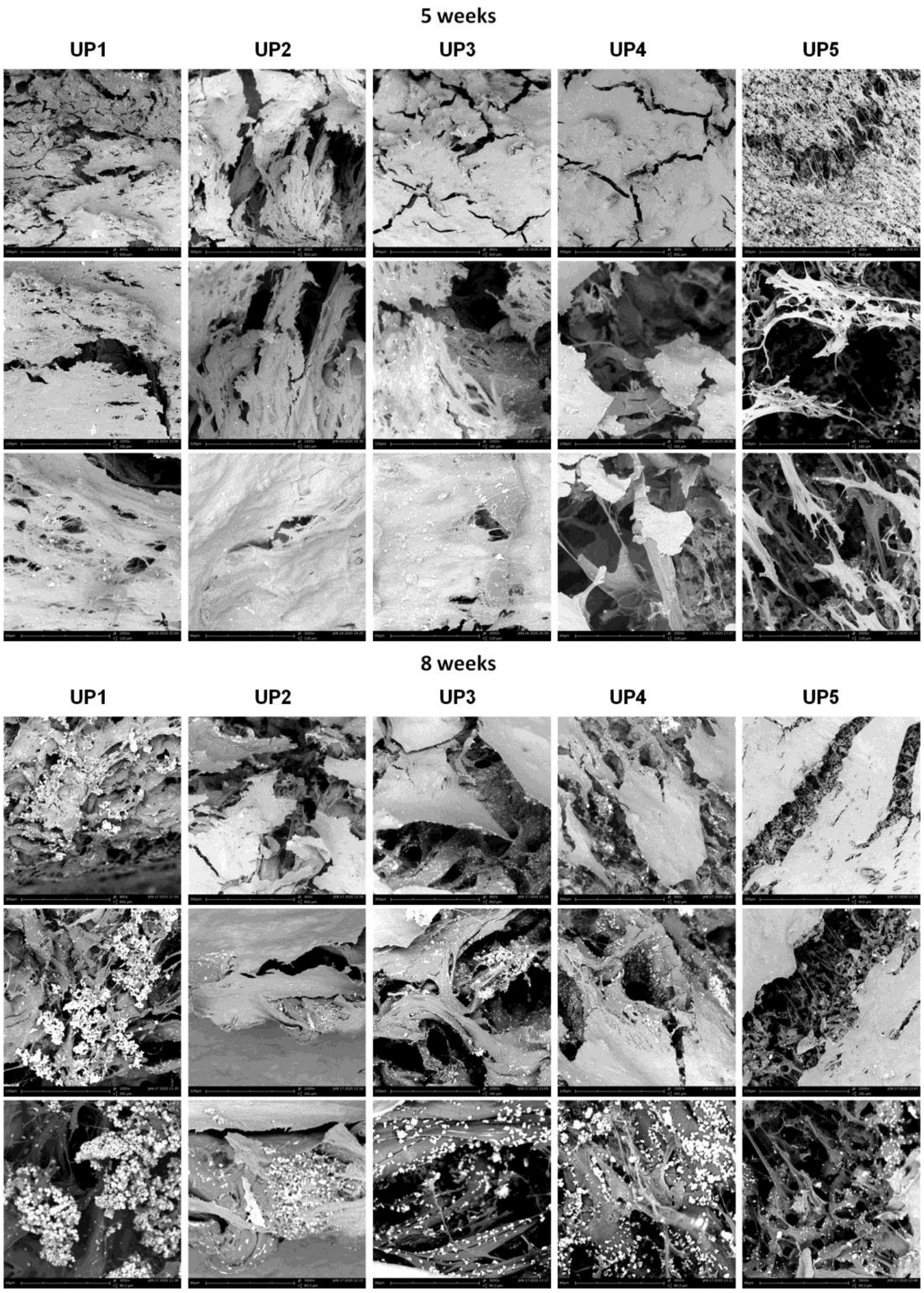

Figure S2. SEM images of UP scaffolds seeded with hADMSCs, depicting the adhesion and spreading of hADMSCs on the porous structure of the scaffolds after 1 week culture in MSC expansion medium and 2, 5 and 8 weeks culture in osteogenic differentiation medium.

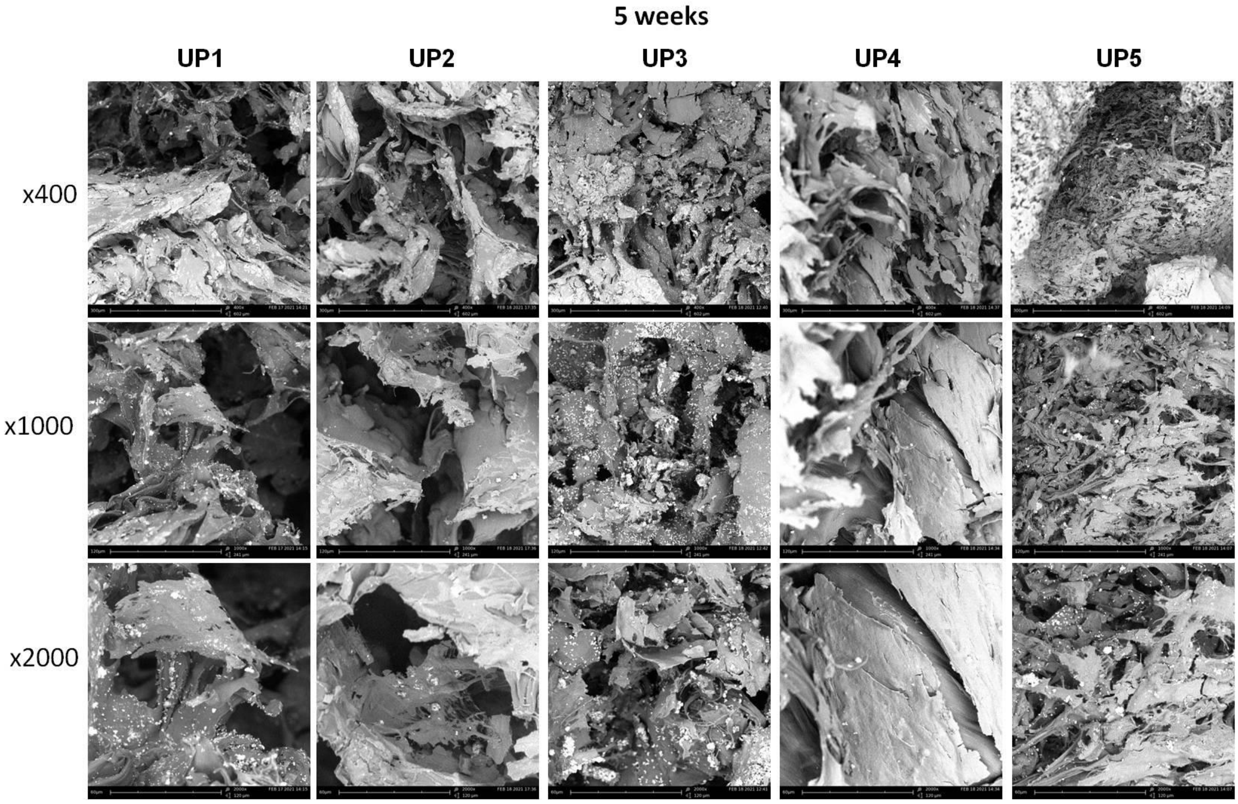

Figure S3. SEM images of cross-sections of the UP scaffolds seeded with hADMSCs, depicting the penetration of hADMSCs in the pores of the scaffolds after 5 weeks culture in osteogenic differentiation medium.
